# Salivary Lactate Dehydrogenase in Relationship to the Severity of Hypoxic-Ischemic Encephalopathy among Newborn Infants

**DOI:** 10.1155/2021/9316277

**Published:** 2021-09-15

**Authors:** Hend Elmoursi, Mohamed Abdalla, Bader Eldin Mesbah, Abdelmoneim Khashana

**Affiliations:** ^1^Faculty of Medicine, Suez Canal University, Ismailia, Egypt; ^2^Clinical Pathology, Faculty of Medicine, Suez Canal University, Ismailia, Egypt; ^3^Pediatrics, Faculty of Medicine, Suez Canal University, Ismailia, Egypt

## Abstract

**Introduction:**

Hypoxic-ischemic encephalopathy (HIE) is defined as a neurological complication that results from perinatal asphyxia. Previous studies had investigated various markers to early detect HIE; however, these markers appeared to have several drawbacks, especially in resource-limited settings.

**Aim:**

This study aimed at evaluating the predictive value of the salivary lactate dehydrogenase level as a potential predictor of hypoxic-ischemic encephalopathy for newborns.

**Materials and Methods:**

We included 30 neonates with HIE due to perinatal asphyxia and 30 healthy newborns that serve as controls, admitted at the intensive care unit for neonates and maternity ward at Ismailia area Clinics and Hospitals. We measured the LDH levels by using saliva samples that were collected for neonates maximum by 12 h after birth.

**Results:**

It was found that patients with HIE had a statistically significant higher salivary LDH level (1927 ± 390.3 IU/L) than patients without HIE (523.6 ± 142.8 IU/L) (*p* < 0.001). Moreover, salivary LDH showed a good discriminative ability where the AUC was 0.966 regarding salivary LDH (95% CI: 0.917–1.0) (*p* < 0.001). The best cutoff value was 1420 IU/L or more which showed the best results in predicting the occurrence of HIE with 98.3% and 97.6% sensitivity and specificity, respectively.

**Conclusion:**

Salivary LDH can be considered as a useful noninvasive laboratory marker that can accurately predict HIE incidence among neonates with asphyxia within 12 hours from birth. The cases in the HIE group were assigned into three stages according to the Sarnat and Sarnat staging system: stage I: mild (irritable, normal, or hypertonia and poor feeding); stage II: moderate (lethargy, hypotonia, and frequent seizure); stage III: severe (coma, flaccid, absent reflexes, and frequent seizure). There is a positive association between LDH levels and the severity of HIE.

## 1. Introduction

Annually, about 4 million newborn children die, and the cause of about a quarter (23%) of these deaths is perinatal asphyxia. Furthermore, the majority of these deaths are in developing countries [1].

Perinatal asphyxia is well known for its progressive damage affecting all the systems and organs, in particular the nervous system; it takes the neurological manifestations up to three days (72 hours) to appear. In born children, the prolonged hypoxic conditions resulting from birth asphyxia may result in impairing blood flow to vital organs including the brain, resulting in brain injury that is manifested by itself as a neurobehavioral state, which is identified as hypoxic-ischemic encephalopathy (HIE) [2].

In order to provide a description of the physical condition of the newborn at birth, the Apgar score is employed. Apgar score's depression may be caused by any hypoxic state, while the Apgar score's prolonged depression is related to the outcome of severe neurodevelopment or death [3].

A large number of studies were conducted as an attempt to relate the perinatal asphyxia's biochemical markers to its neurological consequences and HIE stages [4]. The findings of these studies indicated that these biomolecules, particularly LDH, display a significant increase in the initial perinatal period.

The origin of these enzymes is various organs. ALT, followed by AST, is the most specific enzyme for the liver and is found, for example, in the erythrocytes, muscles, and myocardium, whereas LDH can be found in the majority of the tissues in the body. After the organ damage and asphyxia that occur immediately after birth, a significant increase regarding the levels of LDH, ALT, and AST is observed most probably due to this damage that follows asphyxia. This significant increase as well as their various disappearance rates from plasma (5–36 hours), leads these enzymes to be possible predictors of the hypoxic-ischemic insult's severity regarding the perinatal period [5].

The medical treatment that has long-term neurodevelopmental consequences is therapeutic hypothermia, and the stage of HIE is taken into account when making decisions regarding this treatment should be administered or not [6].

So, in this study with the above context of varied opinions, it is going to investigate if any correlation of lactate dehydrogenase (LDH) with the occurrence of different stages of HIE is present or not and how it is related with different stages of HIE in order to be able to use this enzyme as a marker or predictor of HIE before the appearance of significant clinical features to allow earlier management of HIE and improve the mortality and morbidity rates.

### 1.1. Patients and Methods

This was an observational, cross-sectional study at the intensive care unit for neonates and maternity ward at Ismailia area Clinics and Hospitals. We obtained informed consent from all included neonates' parents prior to any intervention. The ethics committee of clinical research at the Faculty of Medicine, Suez Canal University, approved this study.

### 1.2. Participants

We included newborns within 12 h after birth at the intensive care unit for neonates and maternity ward at Ismailia area Clinics and Hospitals. Two groups of newborns at ≥37 weeks of gestation within 12 h after birth were included. The first group had HIE due to perinatal asphyxia. The second group was composed of healthy neonates that serve as controls.

## 2. Methods

We defined perinatal asphyxia as a need of resuscitation at birth, as well as having one or more of the following:

- Apgar score≤ 5 at 5 min after birth
- Umbilical arterial pH (pHa) < 7.00 mmol/L or base deficit ≥ 16
- More than 10 min of resuscitation of positive pressure ventilation prior to stable spontaneous respiration

We defined HIE as the existence of lethargy or coma along with at least one of the following: abnormal reflexes, including pupillary and oculomotor abnormalities, hypotonia, either weak or absent sucking reflex, abnormal aEEG, and seizures. The neonates with HIE were assigned into three different groups according to the Sarnat and Sarnat staging system:

  Stage I: mild (irritable, normal, or hypertonia and poor feeding)
  Stage II: moderate (lethargy, hypotonia, and frequent seizure)
  Stage III: severe (coma, flaccid, absent reflexes, and frequent seizure)

A total number of 30 neonates were retrospectively enrolled in this study. Neonates who were in need for resuscitation at birth or with a history of delayed cry were assigned to the HIE group, also if they developed HIE within 12 h after birth.

The control group included 30 healthy neonates that serve as controls. We measured the LDH levels by using the saliva samples that were collected within 12 h following birth. Both groups were subjected to detailed history taking, clinical examination, and laboratory investigations including the human lactate dehydrogenase (LDH) ELISA kit.

Exclusion criteria were genetic, syndromic, or other anomalies.

### 2.1. Test Principle

The used kit was a double-antibody sandwich enzyme-linked immunosorbent assay (ELISA) to assess the level of human lactate dehydrogenase (LDH) in samples. We added lactate dehydrogenase (LDH) to the monoclonal antibody enzyme, which was precoated with the human lactate dehydrogenase (LDH) monoclonal antibody and incubated; then, lactate dehydrogenase (LDH) antibodies were labeled with biotin and combined with streptavidin-HRP to form an immune complex; then, we incubated and washed again for the removal of the uncombined enzyme [7].

After that, we added chromogen solution A and B. The color of the liquid changes first into blue, but under the acid's effects, it finally becomes yellow. There was a positive correlation between the chroma of color and the concentration of the human substance lactate dehydrogenase (LDH).

### 2.2. Statistical Analysis

We handled our data using SPSS software, version 16.0. Quantitative data were shown as mean and SD and median and range according to the appropriate measure, while qualitative data were expressed as frequencies and percentages. In parametric data, unpaired *t*-test was used for comparing the quantitative variable between the two independent groups, while we used chi-square test (*x*^2^ value) for comparing the qualitative variable between the two independent groups. Diagrammatic and tabular forms were used when appropriate. Regression was done for the predictors and correlation between the level of salivary lactate dehydrogenase and severity of hypoxic-ischemic encephalopathy. The results were considered significant if the *p* value is less than 0.05.

## 3. Results

This was an observational cross-sectional study that included 60 newborns within 12 h after birth at the intensive care unit and maternity ward of neonates at Ismailia area Clinics and Hospitals. The study participants were divided into two groups: (i) cases: newborns with hypoxic-ischemic encephalopathy (HIE) resulting from perinatal asphyxia and (ii) controls: healthy newborns that serve as controls. The study aimed at evaluating the predictive value of the salivary lactate dehydrogenase level as a potential predictor of hypoxic-ischemic encephalopathy for newborns.

Table 1 shows baseline characteristics of the studied sample. The mean gestational age of HIE patients (37.8 ± 0.78 weeks) was comparable to that of control participants (38.23 ± 1.35) (*p*=0.635). Females formed 53.3% of the HIE patients and 40% of the participants in the control group. Moreover, patients in the HIE group had a lower Apgar score than control participants (*p* < 0.001).

Table 2 shows laboratory measures in both groups. Patients with hypoxic-ischemic encephalopathy had a statistically significant higher salivary LDH level (1927 ± 390.3 IU/L) than patients without hypoxic-ischemic encephalopathy (523.6 ± 142.8 IU/L) (*p* < 0.001).

The level of salivary LDH concerning gender (*p*=0.22) and mode of delivery (*p*=0.56) showed no statistical difference.

Table 3 shows receiver operating characteristic curves for salivary LDH. Salivary LDH showed a good discriminative ability where the AUC for salivary LDH was 0.966 (95% CI: 0.917–1.0) (*p* < 0.001).

Table 4 shows the best cutoff points regarding salivary LDH to predict hypoxic-ischemic encephalopathy in newborns. A cutoff value of 1420 IU/L or more was the best cutoff point to predict the occurrence of HIE with 98.3% and 97.6% sensitivity and specificity, respectively.

We used logistic regression analysis to assess predictors of hypoxic-ischemic encephalopathy among newborns. *R*^2^ = 0.750, where 75% of the variability of the HIE occurrence among newborns can be explained by this model. It was found that, as salivary LDH increases by 100 IU/L, the odds of hypoxic-ischemic encephalopathy occurrence in a term infant increase by 70% (*p*=0.001).

Table 5 presents the comparison between the mean levels of LDH; the data indicate that there is a statistically significant difference in LDH levels according to the severity of hypoxic-ischemic encephalopathy (*p* < 0.001^*∗*^).

## 4. Discussion

In the developing countries, approximately 2.3–26.5 per 1000 live births suffer from hypoxic-ischemic encephalopathy (HIE) annually [8]. However, early manifestations of HIE are unclear subjecting neonates at risk to be diagnosed lately and in turn miss the therapeutic window of six hours [9]. Subsequently, having a routine laboratory marker that can easily predict HIE occurrence among patients at risk is pivotal.

Meanwhile, HIE markers have been extensively studied in order to rescue neonates with perinatal asphyxia to benefit from therapeutic hypothermia [10]. However, these markers appeared to have several drawbacks, especially in resource-limited settings [11]. On the contrary, lactate dehydrogenase (LDH) is well known for its excellent diagnostic accuracy in predicting any tissue damage, especially tissue hypoxia [12].

Drawing LDH samples from saliva seems more convenient than blood sampling; moreover, it can reflect the tissue damage condition as good as the blood sample [13]. Therefore, we aimed to evaluate the predictive value of salivary LDH as a potential predictor of HIE among neonates with perinatal asphyxia.

In the present cross-sectional study, we included two groups. The first group is newborns with HIE due to perinatal asphyxia, with a mean gestational age of 37.8 ± 0.78 weeks and mean birth weight of 3.07 ± 0.45 kg, and the second group serves as controls.

Regarding the salivary LDH level in neonates with and without HIE, we found that patients with HIE had a statistically significant higher salivary LDH level (1927 ± 390.3 IU/L) than patients without HIE (523.6 ± 142.8 IU/L) (*p* < 0.001). This result is consistent with Mehta et al., who found that patients with HIE showed a considerably higher salivary LDH level than healthy controls (2578 vs. 558.5 IU/L) (*p* < 0.001) [14]. Although Mehta et al.'s article was the first and only article, to our knowledge, which tested salivary LDH to predict HIE, many studies have widely used serum LDH to anticipate HIE incidence in newborns with birth asphyxia. For example, Karunatilaka et al. stated that serum LDH was significantly higher in HIE neonates in comparison to both normal controls and asphyxiated neonates without HIE [15]. Similarly, a 3.5-fold increase in serum LDH of HIE patients was observed compared to controls (*p* < 0.001) [12]. Interestingly, Thoresen et al. found that HIE patients treated with hypothermia and who had desirable outcomes reported significantly lower serum LDH than those with tragic events [16]. This proves the sensitivity of LDH in pursuing the track of insult in HIE. This sensitivity can be explained by the release of LDH from the intracellular component of brain tissue after exposure to hypoxia [17]. Consistently, salivary LDH rises in neonates with perinatal asphyxia which is in correlation to serum LDH due to leakage from plasma [18].

Consistently, many articles showed that improved anthropometric measures, including head circumference, from birth to discharge strongly suggest more desirable motor and neurological outcomes later on [19].

Early detection of HIE among patients with asphyxia has been a subject of study in many studies. For example, amplitude-integrated electroencephalography (aEEG) showed high false positive rate during the first 24 hrs after birth [20]. On the contrary, cranial ultrasound showed false reassuring findings that appeared abnormal on MRI later on [21]. MRI is now considered the gold standard tool in diagnosing structural abnormalities related to HIE; however, it is limited to specialized centers and not easily available in all settings [22].

Several biochemical markers have been related to HIE occurrence; however, their diagnostic accuracy in early prediction is indeterminate [22]. On the contrary, salivary and serum LDH showed an excellent discriminative ability in detecting HIE among asphyxiated newborns. Our study reported that the salivary LDH level ≥1420 IU/L was the best cutoff point to predict the occurrence of HIE with a sensitivity of 98.3% and a specificity of 97.6%. Moreover, as salivary LDH increases by 100 IU/L, the odds of HIE occurrence in a term infant increase by 70% (*p*=0.001). Meanwhile, Mehta et al. reported the first cutoff value of salivary LDH for predicting HIE among asphyxiated newborns which was ≥894 IU/L which had 90% and 73.3% sensitivity and specificity, respectively [14]. Karlsson et al. found that serum LDH was the best predictor of HIE with 100% and 97% sensitivity and specificity, respectively, with a cutoff level of 1049 IU/L [12].

Although some evidence showed that LDH is not the optimal biomarker to use in predicting HIE long-term outcomes [15], a later study showed that LDH has 100% sensitivity and 91% specificity in predicting long-term outcomes after HIE when identifying serum LDH at 1176 IU/L [12].

Although the Apgar score cannot accurately predict the neurological consequences of birth asphyxia, yet it is the most clinically accepted and applicable method for assessing postpartum hypoxic-ischemic events [23]. Our results showed that patients in the HIE group had a lower Apgar score than control participants (*p* < 0.001). This is in agreement with all previous articles. Meanwhile, we found that there is a negative association between the Apgar score and salivary LDH among cases (*r* = −0.138). This is consistent with Karlsson et al. who found a negative correlation between the Apgar score at 5 min and serum LDH (*B* = −373; CI: −580, −165) [12].

We believe that our study brings a novel finding regarding using salivary LDH as a more convenient method to predict HIE among neonates at risk. Only one study, to our knowledge, has tested this hypothesis [14]. On the contrary, this study included certain limitations. Our study did not evaluate the value of salivary LDH in predicting long-term sequel of HIE, especially the neurological one.

In conclusion, our study suggests the clinical usefulness of using salivary LDH in predicting HIE incidence in neonates suffering from asphyxia within 12 hrs from birth. However, there is still an urgent need for further research studies where a larger sample size is present in order to obtain a high level of evidence regarding routine use of salivary LDH in neonates with asphyxia to be used as a routine screening test in newborns at risk.

Mehta et al. reported that salivary lactate dehydrogenase levels can provide early diagnosis of hypoxic-ischemic encephalopathy in neonates with birth asphyxia [14].

In this study, we discuss the relationship between the salivary LDH level and the severity of HIE.

## Figures and Tables

**Table 1:** Baseline characteristics of the studied sample.

Variables	All patients (*n* = 60)	Hypoxic-ischemic encephalopathy		*p* value
		Present (*n* = 30)	Absent (*n* = 30)	
*Gestational age (weeks)*				
Mean ± SD	38.15 ± 1.117	38.07 ± 0.83	38.23 ± 1.35	0.635^a^
Median (range)	38 (36–41)	38 (37–39)	38 (36–41)	

*Gender*, *n (%)*				
Male	32 (53.3)	14 (46.7)	18 (60)	0.438^b^
Female	28 (46.7)	16 (53.3)	12 (40)	

*Birth weight (kg)*, *mean* *±* *SD*				
Mean ± SD	2.98 ± 0.537	3.07 ± 0.45	2.90 ± 0.607	0.225^a^
Median (range)	3 (2–4)	3 (2–4)	3 (2–4)	

*Mode of delivery*, *n (%)*				
CS	27 (45)	13 (43.3)	14 (45)	0.795^b^
Vaginal	33 (55)	17 (56.7)	16 (53.3)	

*Apgar score*, *mean* *±* *SD*				
Mean ± SD	6.7 ± 2.78	4.03 ± 0.89	9.37 ± 0.49	0.001^*∗*^^a^
Median (range)	7.5 (3–10)	4 (3–6)	9 (9–10)	

^a^Mann–Whitney test, statistical significance at *p* < 0.05.^b^Chi-square test, statistical significance at *p* < 0.05.

**Table 2:** Comparison of the salivary LDH level between cases and controls.

Variables	All patients (*n* = 60)	Hypoxic-ischemic encephalopathy		*p* value
		Present (*n* = 30)	Absent (*n* = 30)	
Salivary LDH level (IU/L)				
Mean ± SD	1225.3 ± 765.2	1927 ± 390.3	523.6 ± 142.8	0.001^*∗*^^a^
Median (range)	1120 (240–2950)	1840 (1390–2950)	515 (240–850)	

^a^Mann–Whitney test, statistical significance at *p* < 0.05.

**Table 3:** Area under the curve for salivary LDH as a predictor of HIE.

Variable	Area	Stand. error	*p* value	95% CI
Salivary LDH	0.966	0.025	<0.001^*∗*^	0.917–1.0

^∗^Statistical significance < 0.05.

**Table 4:** Sensitivity, specificity, and diagnostic accuracy at different cutoff levels of salivary LDH.

Cutoff points	Sensitivity (%)	Specificity (%)
835	98.3	95.3
1420	98.3	97.6
1560	96.6	97.6

^∗^PPV: positive predictive value; NPV: negative predictive value.

**Table 5:** LDH levels according to the stages of hypoxic ischemic encephalopathy.

LDH level			
Stages of HIE	*N*	Mean ± SD	*p* value
Stage I: mild	6	1500 ± 70.14	<0.001^*∗*^
Stage II: moderate	7	1680 ± 46.90	
Stage III: severe	17	2179.4 ± 333.10	

^*∗*^Full significant as *p* < 0.05_._ Stage I: mild (irritable, normal, or hypertonia and poor feeding); stage II: moderate (lethargy, hypotonia, and frequent seizure); stage III: severe (coma, flaccid, absent reflexes, and frequent seizure).

## Data Availability

The data used to support the findings of this study are included within the article.

## References

[1] Lawn J. E., Cousens S., Zupan J. (2005). 4 million neonatal deaths: when? Where? Why?. *The Lancet*.

[2] Thornton C., Rousset C. I., Kichev A. (2012). Molecular mechanisms of neonatal brain injury. *Neurology Research International*.

[3] Jacobs S. E., Berg M., Hunt R., Tarnow‐Mordi W. O., Inder T. E., Davis P. G. (2013). Cooling for newborns with hypoxic ischaemic encephalopathy. *Cochrane Database of Systematic Reviews*.

[4] Karlsson M., Blennow M., Nemeth A., Winbladh B. (2006). Dynamics of hepatic enzyme activity following birth asphyxia. *Acta Paediatrica*.

[5] Beken S., Aydın B., Dilli D., Erol S., Zenciroğlu A., Okumuş N. (2014). Can biochemical markers predict the severity of hypox-icischemic encephalopathy?. *Turkish Journal of Pediatrics*.

[6] Zhou W. H., Cheng G. Q., Shao X. M. (2010). Selective head cooling with mild systemic hypothermia after neonatal hypoxic-ischemic encephalopathy: a multicenter randomized controlled trial in China. *The Journal of Pediatrics*.

[7] Dawson B. (2004). Methods of evidence-based medicine and decision analysis. *Basic & Clinical Biostatistics*.

[8] Kurinczuk J. J., White-Koning M., Badawi N. (2010). Epidemiology of neonatal encephalopathy and hypoxic-ischaemic encephalopathy. *Early Human Development*.

[9] Sarnat H. B., Sarnat M. S. (1976). Neonatal encephalopathy following fetal distress. *Archives of Neurology*.

[10] Jacobs S. E., Berg M., Hunt R., Tarnow-Mordi W. O., Inder T. E., Davis P. G. (2013). Cooling for newborns with hypoxic ischaemic encephalopathy. *Cochrane Database of Systematic Reviews*.

[11] Murray D. M. (2019). Biomarkers in neonatal hypoxic-ischemic encephalopathy-Review of the literature to date and future directions for research. *Handbook of Clinical Neurology*.

[12] Karlsson M., Wiberg-Itzel E., Chakkarapani E., Blennow M., Winbladh B., Thoresen M. (2010). Lactate dehydrogenase predicts hypoxic ischaemic encephalopathy in newborn infants: a preliminary study. *Acta Paediatrica*.

[13] Aps J. K., Martens L. C. (2005). Review: the physiology of saliva and transfer of drugs into saliva. *Forensic Science International*.

[14] Mehta A., Chawla D., Kaur J., Mahajan V., Guglani V. (2015). Salivary lactate dehydrogenase levels can provide early diagnosis of hypoxic-ischaemic encephalopathy in neonates with birth asphyxia. *Acta Paediatrica*.

[15] Karunatilaka D. H., Amaratunga D. S., Perera N. I., Caldera V. (2000). Serum creatine kinase and lactic dehydrogenase levels as useful markers of immediate and long-term outcome of perinatal asphyxia. *Sri Lanka Journal of Child Health*.

[16] Thoresen M., Liu X., Jary S. (2012). Lactate dehydrogenase in hypothermia-treated newborn infants with hypoxic-ischaemic encephalopathy. *Acta Paediatrica*.

[17] Naithani M., Singh P. (2006). Teitz textbook of clinical chemistry & molecular diagnostics. *Medical Journal Armed Forces India*.

[18] Miller C. S., King C. P., Langub M. C., Kryscio R. J., Thomas M. V. (2006). Salivary biomarkers of existing periodontal disease. *The Journal of the American Dental Association*.

[19] Ehrenkranz R. A., Dusick A. M., Vohr B. R., Wright L. L., Wrage L. A., Poole W. K. (2006). Growth in the neonatal intensive care unit influences neurodevelopmental and growth outcomes of extremely low birth weight infants. *Pediatrics*.

[20] Gluckman P., Wyatt J., Azzopardi D. (2005). Selective head cooling with mild systemic hypothermia after neonatal encephalopathy: multicentre randomised trial. *The Lancet*.

[21] Van Laerhoven H., De Haan T. R., Offringa M., Post B., Van Der Lee J. H. (2013). Prognostic tests in term neonates with hypoxic-ischemic encephalopathy: a systematic review. *Pediatrics*.

[22] Merchant N., Azzopardi D. (2015). Early predictors of outcome in infants treated with hypothermia for hypoxic-ischaemic encephalopathy. *Developmental Medicine and Child Neurology*.

[23] Antonucci R., Porcella A., Pilloni M. D. (2014). Perinatal asphyxia in the term newborn. *Journal of Pediatric and Neonatal Individualized Medicine*.

